# Supplementary material for: Modelling the impact of decidual senescence on embryo implantation in human endometrial assembloids
Source: eLife. 2021 Sep 6;10:e69603. doi: 10.7554/eLife.69603 (PMC8523170; doi:10.7554/eLife.69603)
Supplement: Transparent reporting form [file elife-69603-transrepform1.docx]

***eLife’s* transparent reporting form**

We encourage authors to provide detailed information *within their submission* to facilitate the interpretation and replication of experiments. Authors can upload supporting documentation to indicate the use of appropriate reporting guidelines for health-related research (see [EQUATOR Network](http://www.equator-network.org/%20)), life science research (see the [BioSharing Information Resource](https://biosharing.org/)), or the [ARRIVE guidelines](http://www.plosbiology.org/article/info:doi/10.1371/journal.pbio.1000412) for reporting work involving animal research. Where applicable, authors should refer to any relevant reporting standards documents in this form.

If you have any questions, please consult our Journal Policies and/or contact us: [editorial@elifesciences.org](mailto:editorial@elifesciences.org).

**Sample-size estimation**

- You should state whether an appropriate sample size was computed when the study was being designed
- You should state the statistical method of sample size computation and any required assumptions
- If no explicit power analysis was used, you should describe how you decided what sample (replicate) size (number) to use

Please outline where this information can be found within the submission (e.g., sections or figure legends), or explain why this information doesn’t apply to your submission:

No statistical methods were used to determine sample size, we followed standards in the field when using primary human tissue and according to sample availability. Sample size for each experiment was always at least 3 biological replicates and is stated in the results section and figure legend, where appropriate.

**Replicates**

- You should report how often each experiment was performed
- You should include a definition of biological versus technical replication
- The data obtained should be provided and sufficient information should be provided to indicate the number of independent biological and/or technical replicates
- If you encountered any outliers, you should describe how these were handled
- Criteria for exclusion/inclusion of data should be clearly stated
- High-throughput sequence data should be uploaded before submission, with a private link for reviewers provided (these are available from both GEO and ArrayExpress)

Please outline where this information can be found within the submission (e.g., sections or figure legends), or explain why this information doesn’t apply to your submission:

Biological replicates, defined as independent patients, are indicated in the text and figure legends for each experiment. Patient information is provided in Supplementary Table 2. Assembloids from N=3 patient biopsies were subjected to differentiation protocols in Figure 1. A further N=3 independent biopsies were used for the minimal differentiation media experiments in Figure 2. Single-cell RNA sequencing (scRNAseq) was performed on assembloids generated from a further N=3 independent patient samples. Quality control steps performed in single cell RNA sequencing (scRNAseq) analysis to remove low quality (<200 captured genes) or dead/dying cells (>5% mitochondrial reads) are described in the methods section and followed standard workflows. scRNAseq data has been uploaded to Gene Expression Omnibus (ncbi.nlm.nih/gov/geo; GSE168405). Four independent patient samples were used for ELISA examination in Figure 5. Ten single embryos were used for co-culture in assembloids generated from two independent patient samples, in Figure 6. Figure 6-figure supplement 1 shows representative images from one patient in an experiment of N=3 patients.

**Statistical reporting**

- Statistical analysis methods should be described and justified
- Raw data should be presented in figures whenever informative to do so (typically when N per group is less than 10)
- For each experiment, you should identify the statistical tests used, exact values of N, definitions of center, methods of multiple test correction, and dispersion and precision measures (e.g., mean, median, SD, SEM, confidence intervals; and, for the major substantive results, a measure of effect size (e.g., Pearson's r, Cohen's d)
- Report exact p-values wherever possible alongside the summary statistics and 95% confidence intervals. These should be reported for all key questions and not only when the p-value is less than 0.05.

Please outline where this information can be found within the submission (e.g., sections or figure legends), or explain why this information doesn’t apply to your submission:

Statistical analysis methods are presented in the Methods and Materials section, and stated in the relevant figure legends, along with definition of centre and error, and details of multiple testing corrections where appropriate. Individual data points are presented in figures and available in the source data file as indicated.

(For large datasets, or papers with a very large number of statistical tests, you may upload a single table file with tests, Ns, etc., with reference to sections in the manuscript.)

**Group allocation**

- Indicate how samples were allocated into experimental groups (in the case of clinical studies, please specify allocation to treatment method); if randomization was used, please also state if restricted randomization was applied
- Indicate if masking was used during group allocation, data collection and/or data analysis

Please outline where this information can be found within the submission (e.g., sections or figure legends), or explain why this information doesn’t apply to your submission:

Experiments on endometrial samples were internally controlled, i.e. conditions were applied to parallel wells established from the same biopsy/cells. For embryo co-culture experiments, thawed embryos were pooled before being transferred at random to assembloid wells derived from a single endometrial biopsy per experiment.

**Additional data files (“source data”)**

- We encourage you to upload relevant additional data files, such as numerical data that are represented as a graph in a figure, or as a summary table
- Where provided, these should be in the most useful format, and they can be uploaded as “Source data” files linked to a main figure or table
- Include model definition files including the full list of parameters used
- Include code used for data analysis (e.g., R, MatLab)
- Avoid stating that data files are “available upon request”

Please indicate the figures or tables for which source data files have been provided:

Source data have been provided as follows:

Figure 1D, ELISA data – Figure 1 Source Data 1

Figure 2, PCR data – Figure 1 Source Data 1

Figure 3C, Epithelial sub-population markers – Figure 3 Source Data 1

Figure 3D and F, GO analysis results – Figure 3 Source Data 2

Figure 3E, Stromal sub-population markers – Figure 3 Source Data 3

Figure 4, CellPhoneDB predicted ligand-receptor interactions – Figure 4 Source Data 1

Figure 5C, Differentially expressed genes after dasatinib treatment – Figure 5 Source Data 1

Figure 5, GO analysis results – Figure 5 Source Data 2

Figure 5D, ELISA data – Figure 5 Source Data 3

Figure 6D, Embryo diameters – Figure 6 Source Data 1

Figure 6F, Embryo hCG secretion – Figure 6 Source Data 2
